# Lubricant-entrenched slippery surface-based nanocarriers to avoid macrophage uptake and improve drug utilization

**DOI:** 10.1016/j.jare.2022.08.015

**Published:** 2022-08-28

**Authors:** Chengduan Yang, Jianming Feng, Ziqi Liu, Juan Jiang, Xiafeng Wang, Cheng Yang, Hui-jiuan Chen, Xi Xie, Liru Shang, Ji Wang, Zhenwei Peng

**Affiliations:** a The First Affiliated Hospital of Sun Yat-Sen University, Sun Yat-Sen University, Guangzhou, China; b State Key Laboratory of Optoelectronic Materials and Technologies, School of Electronics and Information Technology, Sun Yat-Sen University, Guangzhou, China

**Keywords:** Nanocarriers, Lubricant-entrenched slippery surfaces, Anti-adhesion of proteins, Anti-uptake of macrophage, Improving drug utilization

## Abstract

**Introduction:**

Reducing the protein adsorption of nanoparticles (NPs) as drug carriers to slow their rapid clearance by macrophages uptake is a critical challenge for NPs clinical translational applications. Despite extensive research efforts to inhibit cellular uptake, including covering biological agents or surface chemical coatings to impart “stealth” properties to NPs, their stability remains insufficient.

**Objectives:**

Developed a novel surface modification technology based on a physical infusion engineering approach to achieve persistent inhibition of protein adhesion and cellular uptake by nanocarriers.

**Methods:**

The nanoparticles were prepared based on conventional drug carrier mesoporous silica NPs through a two-step process. A functional nanoscale slippery surface was formed by grafting “liquid-like” brushes on the particles surface, and then a lubricant-entrenched slippery surfaces (LESS) was formed by infusing silicone oil lubricant into the entire surface. Co-incubation with macrophages (*in vitro* and *in vivo*) was used to examine the anti-uptake properties of modified NPs. The anti-adhesion properties of LESS coating surfaces to various liquids, proteins and cells were used to analyze the anti-uptake mechanism. Loaded with drugs, combined with tumor models, to evaluate the drug utilization of modified NPs.

**Results:**

Relying on the stable and slippery LESS coating, the modified surface could prevent the adhesion of various liquids and effectively shield against the adhesion of proteins and cells, as well as remarkably reduce macrophage cellular uptake *in vitro* and *in vivo*. In addition, the LESS coating does not affect cell activity and allows NPs to be loaded with drugs, significantly improving the utilization of drugs *in vitro* and *in vivo*. This allows the NPs to reach to the target tumor site for drug delivery without active clearance by macrophages.

**Conclusion:**

Our research introduces a new nanocarrier technology to improve anti-biofouling performance and stealth efficiency that will facilitate the development of nanomedicines for clinical transformation applications.

## Highlights

• Physical infusion-based lubricant-entrenched slippery nanoparticles were designed to resist nonspecific uptake.
• The anti-uptake properties of the lubricant-entrenched slippery nanoparticles were achieved by the formation of a slippery surface to resist the adhesion of proteins and cells.
• Lubricant-entrenched slippery nanoparticles remarkably reduced macrophage cellular uptake *in vitro* and *in vivo*. Moreover, excellent biocompatibility was exhibited, and the utilization of drugs *in vitro* and *in vivo* was significantly improved.

## Graphical abstract

**Figure:**
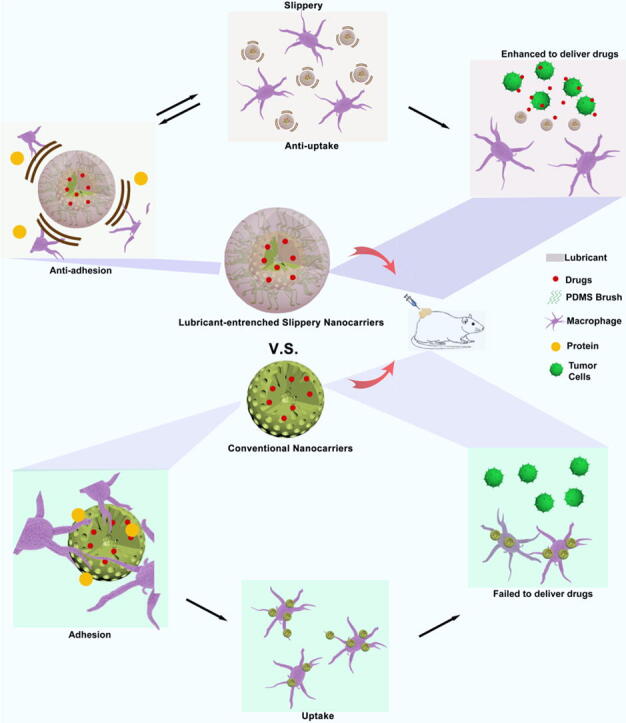

## Introduction

For decades, bionanomaterials including nanoelectrodes, nanocellulose, nanopore materials (metal–organic frameworks (MOFs) and others), and nanorobots have been developed for the diagnosis and treatment of various human diseases[1], [2], [3], [4], [5], [6]. Moreover, through bionanomaterials engineering (to produce tailored physicochemical properties), more effective and safer disease diagnostic and treatment methods have been developed, making biological/clinical medical applications possible[7], [8]. In particular, the use of typical bionanomaterials and nanoparticles as drug carriers is considered to be one of the most promising tools for the treatment of human diseases (such as cancer) and shows great potential in applications such as nanomedicine drug delivery[9], [10]. Despite tremendous progress in fundamental research, these nanoparticles are susceptible to biological barriers (e.g., the mononuclear phagocytic system) in complex body-fluid environments, which significantly limits the delivery efficiency of nanoparticles to release drugs into targeted diseased tissues[11], [12]. On the other hand, most of the administered dose is distributed to organs (such as the liver and spleen) with abundant macrophage accumulation because of biological barriers[13], which can cause severe damage to normal tissues and organs, leading to huge challenges in the clinical transformation of nanomedicine. Therefore, to achieve the clinical translation of nanoparticles for drug delivery, it is imperative to develop an effective method to reduce the interaction between nanoparticles and the phagocytic system, thereby extending the blood circulation time of the nanoparticles and improving drug delivery efficiency.

Current attempts to prevent cell phagocytosis rely on imparting biological properties to nanoparticles to change their biological fate[14], thereby prolonging their blood circulation time. For example, a biomimetic drug delivery system coated with cell membranes or platelets effectively inhibits macrophage uptake due to its surface immunosuppressive properties[15], [16], [17]. However, when biological agents such as red blood cells or cell membranes are used as camouflage materials, high activity of these biological agents must be maintained. In biofluids, the adsorption of proteins on nanoparticles surface forms a corona layer (protein corona) containing opsonins and complement proteins, which facilitate the recognition of nanoparticles by macrophages, thereby initiating rapid clearance of nanoparticles from the blood circulation[18]. Therefore, various anti-biological fouling polymer coatings have been developed to reduce the interaction between nanoparticles and phagocytes by inhibiting the adhesion of proteins, thereby realizing the so-called “stealth” behavior of nanoparticles[18], [19], [20], [21]. For example, due to the hydrophilicity and conformation of the molecular chain, polyethylene glycol (PEG) conjugated onto the surface of a nanocarrier reduces the interaction between the nanomedicine and the immune system, and hence, its uptake by the phagocytic system is inhibited[22], [23]. However, the inherent amphiphilic nature of PEG promotes its nonspecific adsorption of proteins[24], which can induce immune responses such as accelerated blood clearance and pseudoallergic reactions and greatly compromise the PEGylation function[25], [26], [27], [28]. Various polymers including poly(amino acids), poly(glycerol), polybetaines (zwitterionic polymers), poly(acrylamides), and glycopolymers have been investigated as possible alternatives to PEG for various biomedical applications[29]. For example, polybetaine-based zwitterionic polymers exhibit attractive low-fouling properties[30]. However, many of these polymers generally exhibit higher levels of biofouling compared to PEG. On the other hand, a surface that persistently resists proteins is difficult to obtain by relying on only covalent grafting due to molecule oxidation and degradation[31]. Techniques to functionalize nanocarriers and nanoparticles with persistent protein resistance and low uptake properties are in great demand yet difficult to achieve.

Alternatively, a slippery lubricant infusion porous substrate (SLIPS) has been demonstrated as a highly promising anti-biofouling strategy since the lubricating coating is immiscible with various liquids and exhibits resistance to biologically active substances (such as proteins, bacteria, and cells)[31], [32], [33], [34], [35]. Especially for medical materials, such as the surfaces of catheters[36] or microspheres[37], SLIPS have been developed to prevent biofouling and show excellent performance against protein adhesion. Despite its effective antiadhesion ability, the lubricant is easily removed from the surface and causes functional failure in a fluid environment[38], [39], which are characteristics not conducive to application in the living body. Recently, lubricant-entrenched slippery surfaces (LESSs) have been developed and are considered an emerging type of anti-fouling coating[40]. A functionalized slippery surface is formed on the substrate by coating with brush-like molecules with a thin layer of lubricant stabilized by intermolecular forces, and the result exhibits excellent and robust antiadhesion performance in a fluid environment. Compared with polymer grafting, the surface with the LESS strategy showed excellent anti-adhesion properties against blood and bacteria[41], and even against highly viscoelastic solids such as feces[40]. At present, the development and application of LESS coatings are still very limited, especially in the field of biomedicine.

**Fig. 1:**
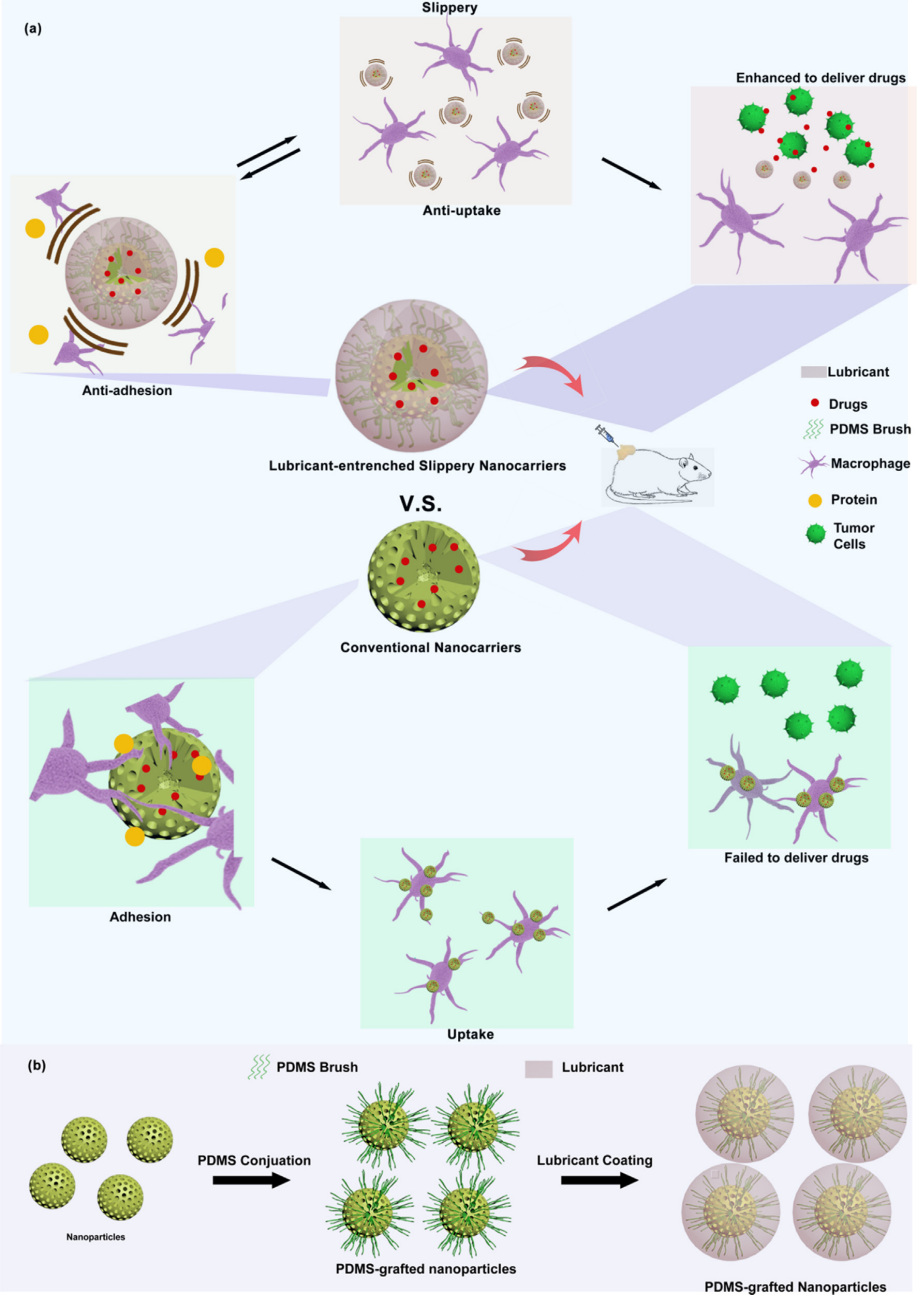
(a) Schematic diagram of the lubricant-entrenched slippery nanoparticles with uptake-resistance, compared to conventional microparticles engulfed by Macrophage. (b) Schematic of the fabrication procedure of the lubricant-entrenched slippery nanoparticles.

Inspired by LESSs, for the first time to our knowledge, lubricant-entrenched slippery nanoparticles (LESNPs) based on a physical infusion engineering method were developed to reduce nonspecific cellular uptake (Fig. 1a). In our design, the nanoparticles were prepared based on conventional drug carrier mesoporous silica nanoparticles through a two-step process. A “liquid-like” polydimethylsiloxane (PDMS) brush was conjugately tethered onto the nanoparticles to form a functional nanoscale slippery surface, and then a silicone oil lubricant with strong chemical affinity was infused into the entire surface to form a highly slippery coating for an anti-biofouling effect. The liquid-entrenched slippery surface exhibited adhesion-resistance properties toward various liquids, as well as proteins and cells, which would generally adhere to phagocytic systems *in vivo*. LESNPs showed excellent resistance to uptake by macrophages *in vitro* and *in vivo* and exhibited biocompatibility without affecting cell viability. On the other hand, conventional nanoparticles without LESS modification underwent heavy macrophage uptake. In addition, our results emphasized that the LESNP-based method could significantly improve drug utilization *in vitro* and *in vivo*. The LESS coating modification technology applied to nanoparticles not only serves as an effective anti-adhesion strategy but also provides a convenient and practical method for achieving an anti-uptake effect in nanoparticles, which opens new avenues for the development of advanced biomedical nanocarriers to achieve clinical transformation.

## Materials and methods

### Fabrication of slippery nanoparticles (SNPs)

Mesoporous silica nanoparticles (∼500 nm mean diameter, Sigma-Aldrich) were used as the base particles. The 200 mg NPs were first cleaned by piranha solution and further functionalized with 1 mL pure OH-PDMS-OH (viscosity 25 cSt, Sigma-Aldrich) at 120 °C for 24 h. After reaction, the NPs were collected by centrifugation at 12,000 rpm for five minutes, washed with toluene to remove excess PDMS, and dried under 60 °C.

### Fabrication of lubricant-entrenched slippery nanoparticles (LESNPs) and lubricant-coated nanoparticles (LCNPs)

The 100 mg slippery nanoparticles were incubated with 0.5 mL liquid lubricant, polydimethylsiloxane (OH-PDMS-OH, viscosity 25 cSt, Sigma-Aldrich) overnight to absorb liquid on the particles and pores surface. Separate the particles and excess liquid lubricating fluid by centrifugation at 12,000 rpm for five minutes, and resuspend the particles in deionized water after remove the upper lubricating medium. The NPs were centrifuged again at 12,000 rpm for five minutes and washed to completely remove unabsorbed liquid lubricant. Similarly, the lubricant-coated nanoparticles (LCNPs) were prepared by the similar procedure using original nanoparticles as base particles.

### Fabrication of nanoparticles with fluorescence

The DI water or liquid lubricant (polydimethylsiloxane (OH-PDMS-OH, viscosity 25 cSt, Sigma-Aldrich)) were first staining with Rhodamine B, and then incubated with nanoparticles and smooth nanoparticles overnight. The different fluorescence nanoparticles were obtained by centrifugation and washing with deionized water.

### Fabrication of lubricant-entrenched slippery glass (LESG) substrate and lubricant-coated glass (LCG) substrate

Similar to LESNPs, the lubricant-entrenched slippery glass (LESG) substrate was prepared by first grafting “liquid-like” PDMS brush onto a substrate to form a smooth surface, followed by the addition of a liquid lubricant. Specifically, glass slides as the substrates were first cleaned by piranha solution and further functionalized with OH-PDMS-OH (viscosity 25 cSt, Sigma-Aldrich) at 120 °C for 24 h. After reaction, the glass slides were washed with toluene to remove excess PDMS and dried under 60 °C. Subsequently, the smooth substrates were incubated with liquid lubricant, polydimethylsiloxane (OH-PDMS-OH, viscosity 25 cSt, Sigma-Aldrich) overnight to absorb liquid on the glass slides surface. Remove excess PDMS liquid by centrifugation and washing with deionized water three times. Similarly, the lubricant-coated glass (LCG) substrate was prepared by directly immersing the ungrafted glass slide in the lubricant through the same procedure.

### Characterization of particles

The morphologies of nanoparticles and PDMS-grafted nanoparticles were visualized by scanning electron microscopy (SEM, Zeiss SUPRA 60) and transmission electron microscopy (TEM, Bruker, Germany). The surface elements analysis of PDMS grafted with glass slide instead of nanoparticles were characterized by X-ray photoelectron spectroscopy (XPS, ESCALAB250Xi). The coating of LESNPs was characterized with optical microscopy or fluorescence microscopy (MF52-LED, Mshot) and Thermogravimetric analysis (TGA, TGA Q500). Nanoparticles size was characterized with Dynamic light scattering (DLS) experiments, which was performed at 25 °C using a Malvern Zetasizer NanoZS instrument.

### Contact angle and dynamic slippery behavior measurement

Static contact angle analysis was characterized with Goniometer measuring system to analyze the wetting properties of the different substrates at room temperature. DI water, ethanol, toluene and Dulbecco’s Modified Eagle Medium(DMEM) with foetal bovine serum (FBS) were used as probe liquids. To test the dynamic slippery behavior of DMEM droplets on different substrates, all substrates including unmodified pristine glass (PG), PDMS chains grafted slippery glass (SG), lubricant coated glass (LCG) and lubricant-entrenched slippery glass (LESG) were tilted with an angle (SA) of 5°. 5 μL droplet was applied to each substrate surface, and the time–sequence images were captured by the Goniometer measuring system.

### Tests of the adhesions of proteins

The different substrates (PG, SG, LCG and LESG) were incubated with 0.1 mg/mL (in PBS, pH 7.4) FITC conjugated bovine serum albumin (FITC-BSA, MW ∼ 66 kDa, Beijing BioDee Biotechnology) and fluorescent fibrinogen (Fg, Solarbio) at 37 °C for 24 h, respectively. After the incubation was completed, all substrates were then washed with fresh PBS for one time. Typical substrates images were recorded with fluorescence microscopy (MF52-LED, Mshot) at the same exposure time, and the relative fluorescence intensity of different substrates surface was quantitatively analyzed by Image J.

### Tests of the adhesion and viability of cells

NIH/3T3 cells and RAW264.7 cells (The First Affiliated Hospital of Sun Yat-Sen University) were first cultured in medium with 90 % DMEM (Dulbecco’s modified Eagle medium, Gibco), 9 % FBS (foetal bovine serum, Gibco) and 1 % antibiotics at 37 °C, 95 % humidity and 5 % CO_2_, respectively. The two types cells (10^5^ ∼ 10^6^ cells / ml) were incubated with different substrates (PG, SG, LCG and LESG) at 37 °C for 24 h, respectively. Prior to imaging, the cells were labeled with 10 µg/ml green-fluorescent dye (Calcein AM, labeling of living cells, Thermo Fisher Scientific, USA) and red fluorescent dye (propidium iodide (PI) labeling of dead cells, Thermo Fisher Scientific, USA). The samples were then imaged with fluorescence microscopy. The number of fluorescent cells anchoring on substrates were counted. The cells viability was quantified by counting the number percentage of both live cells and dead cells.

### Tests of LESNPs uptake by Raw264.7 cells

RAW264.7 cells (1 × 10^5^ cells/per well) were seeded in the 6-well plates and cultured at 37 °C, 95 % humidity and 5 % CO_2_ for 24 h. Afterwards, the cells were incubicated with different fluorescence nanoparticles (NPs, SNPs, LCNPs and LESNPs) and incubated for indicated time periods (e.g. 2 h, 12 h or 24 h). After each time period, the cells were washed with PBS for 3 times and observed by fluorescence microscopy system. For quantitative analysis, the cells were stained with Calcein AM, and subjected to confocal fluorescence microscope (Olympus FV-10i, Zeiss LSM or Leica TCS SP8) and flow cytometry (BD LSRFORTESSA X-20). For confocal fluorescence images, the number of red fluorescent particles taken up into green fluorescent cells was counted. For flow cytometry analysis, quantified with fluorescence signal.

### Tests of LESNPs uptake by macorphages *in vivo*

Peritoneal macrophages of mice were first elicited by intraperitoneal injection (i.p.) of 3 % Thioglycollate Broth (Sigma). After 3 days, the fluorescence labeled nanoparticles (NPs and LESNPs) and PBS solution were injected into the intraperitoneal cavity. After 24 h of injection, macrophages were harvested via peritoneal lavage with 5 mL cold PBS solution. Then, cells were washed with PBS solution and the uptake of different fluorescence nanoparticles were analyzed by flow cytometry.

### Tests of LESNPs uptake by 4T1 cells

4T1 cells (The First Affiliated Hospital of Sun Yat-Sen University) were first cultured in medium with 90 % RPMI 1640 (Roswell Park Memorial Institute, Gibco), 9 % FBS (foetal bovine serum, Gibco) and 1 % antibiotics at 37 °C, 95 % humidity and 5 % CO_2_, respectively. Then 4T1 cells (1 × 10^5^ cells/per well) were seeded in the 24-well plates and cultured at 37 °C, 95 % humidity and 5 % CO_2_ for 24 h. Afterwards, the cells were incubicated with different fluorescence nanoparticles (NPs, SNPs, LCNPs and LESNPs) and incubated for indicated time periods (e.g. 2 h, 12 h or 24 h). After each time period, the cells were washed with PBS for 3 times and observed by fluorescence microscopy system. In addition, the uptake particles by cells were quantified with fluorescence signal by flow cytometry.

### Tests of drug release behavior of LESNPs

The drug release behavior test of LESNPs was completed by two processes: drug loading and drug release. For drug loading, 0.1 mg of the hydrophilic drug Rhodamine B and the hydrophobic drug of Nile Red were dissolved in 1 mL of PDMS liquid respectively, and then 5 mg of slippery particles were added to the above two solutions for incubation overnight. The drug-loaded nanoparticles were collected by centrifugation and water washing, and dispersed in 0.5 mL PBS buffers (0.01 M, pH = 7.4). For drug release, 0.5 mL LESNPs-Rhodamine B and Nile Red were added into a dialysis tube (10 K MWCO) (Slide-A-Lyzer, Thermo Scientific) embedded into 5 mL of the PBS buffer (0.01 M, pH = 7.4), and gently shook in a shaker (QILINBEIER, China) at 100 rpm at 37 °C. At predetermined time intervals, remove the total buffer solution and replace it with 5 mL of the same fresh buffer solution. The characteristic peak absorbance intensity of drugs released was measured by a microplate reader (Rhodamine B ∼ 554 nm, Nile Red ∼ 553 nm).

## Dox loaded onto different nanoparticles

25uL Dox solution (4 mg mL^−1^ in DMF solution) was separately miscible with 500 uL deionized water or PDMS, 5 mg nanoparticles including NPs and SNPs were dispersed in the above two mixtures overnight, and then each drug-loaded particle (NPs-Dox, LCNPs-Dox and LESNPs-Dox) was collected by centrifugation at 12,000 rpm for five minutes and water washing. Measure the absorbance of the characteristic peak of Dox (480 nm) by a microplate reader, the loading percentage of Dox in different nanoparticles was estimated by subtracting the amount of Dox in the collected supernatant from the total amount of Dox added.

### Dox released from different nanoparticles

Similar to the tests of LESNPs-Rhodamine B drug release, Dox-loaded different nanoparticles (NPs-Dox, LCNPs-Dox and LESNPs-Dox) were first dispersed in 0.5 mL PBS buffers (0.01 M, pH = 7.4). and then added into a dialysis tube (10 K MWCO) (Slide-A-Lyzer, Thermo Scientific) embedded into 5 mL of the PBS buffer (0.01 M, pH = 7.4), and gently shook in a shaker (QILINBEIER, China) at 100 rpm at 37 °C. At predetermined time intervals, remove the total buffer solution and replace it with 5 mL of the same fresh buffer solution. The characteristic peak absorbance intensity of Dox released was measured by a microplate reader (480 nm).

### In vitro cytotoxicity

HeLa cells, MCF7 cells and 4T1 cells (The First Affiliated Hospital of Sun Yat-Sen University, 5 × 10^3^ cells / well) were seeded in the 96-well plates and cultured at 37 °C, 95 % humidity and 5 % CO_2_ for 24 h, respectively. Afterwards, the cells were treated with NPs, NPs-Dox, LESNPs and LESNPs-Dox with different NPs/Dox concentrations and incubated for 24 h. Then the cell viabilities were analyzed by a standard live/dead cell staining assay and the 3-(4,5-dimethyl thiazol-2-yl)-2,5-diphenyltetrazolium bromide (MTT) assay. For a standard live/dead cell staining assay, the cells were labeled with 10 µg/ml green-fluorescent dye (Calcein AM, labeling of living cells, Thermo Fisher Scientific, USA) and red fluorescent dye (propidium iodide (PI) labeling of dead cells, Thermo Fisher Scientific, USA). The cells were then imaged with fluorescence microscopy. For MTT assay, the cells were added with MTT solution (20 μL, 5 mg/mL). After 4 h incubation, the medium was replaced with dimethyl sulfoxide (DMSO, 150 μL). The absorbance was measured at the wavelength of 570 nm by a microplate reader.

### Relative cell viability *in vitro*

RAW264.7 cells and 4T1 cells (The First Affiliated Hospital of Sun Yat-Sen University, 5 × 10^3^ cells / well) were seeded in the 96-well plates and cultured at 37 °C, 95 % humidity and 5 % CO_2_ for 24 h, respectively. Afterwards, the RAW 264.7 cells were first treated with NPs-Dox, LCNPs-Dox and LESNPs-Dox with Dox concentrations at 2000 ng / mL and incubated for 2 h. Subsequently, the drugs were removed and added to 4T1 cells and incubated for 24 h, and RAW264.7 cells were continued to be cultured in fresh medium for 22 h. In addition, there is a group of RAW264.7 cells and 4T1 cells without any treatment as a control group. After incubation, the all cells were labeled with 10 µg/ml Calcein AM to record living cells. Imaged cells with fluorescence microscopy and counted green fluorescent cells. The relative cells viability was quantified by the ratio of the relative cell viability of RAW264.7 cells to the relative cell viability of 4T1 cells in each group. While the relative cell viability of RAW264.7 cells was determined by the ratio of the number of green fluorescent RAW264.7 cells in each group to the number of green fluorescent RAW264.7 cells in the control group. And the relative cell viability of 4T1 cells was determined by the ratio of the number of green fluorescent 4T1 cells in each group to the number of green fluorescent 4T1 cells in the control group.

### Antitumor efficacy assay *in vivo*

All research and animal experiments described herein were carried out in accordance with the Animal Care and Use Committee at the Sun Yat-sen University. Wild-type BalB/c mice (female, 6-week old) were all of specific pathogen-free (SPF) grade and obtained from the Animal Facility of Sun Yat-Sen University. The mice were housed under SPF standard conditions at the Animal Experimental Center of Sun Yat-sen University and free fed a standard sterile pellet diet and water.

To obtain 4T1 tumor-bearing mice model, the female nude mice were injected 5 × 10^5^ 4T1 cells in 100 µL saline in the mammary fat pad. When the volume of tumors reached to 600 mm^3^, twenty-eight tumor-bearing mice were randomly divided into four groups (n = 7) and weighed. From day 0, the mice were intratumoral injected with LESNPs-Dox (NPs 400 mg / kg, Dox 2 mg / kg), NPs-Dox, free Dox, and and saline as a control every-two days for 10 days. The tumor size and body weight were measured twice weekly. At day 14, the mice were euthanized by excess intraperitoneal injection of 3 % sodium pentobarbital solution. Collect tumors and measure their actual weight and size separately.

### Statistical analysis

T-test was utilized to analyze the difference between two groups, and one way ANOVA among multiple groups. All data were presented as Mean ± s.e.m.

## Results and discussion

### Preparation and characterization of LESNPs

The fabrication procedure used for lubricant-entrenched slippery nanoparticles (LESNPs) is illustrated in Fig. 1b. It was mainly completed through a two-step process of chemical reaction and coating. The first step formed a smooth brush layer interface on the particle surface by covalent bonding, and the second step formed an outer lubricant layer by coating. In brief, a conventional nanocarrier—mesoporous silica nanoparticles as representative particles—was first washed with piranha solution to remove surface contaminants, and the hydroxyl groups were exposed, which made the surface easy to functionalize. After cleaning and drying, the particles were dispersed in pure low-viscosity (25 cSt) liquid hydroxyl-terminated polydimethylsiloxane (HO-PDMS-OH) and incubated statically at 120 °C for 24 h. After the reaction was completed, the particles were washed with toluene to remove excess PDMS. These molecules underwent a hydrolysis reaction with hydroxyl groups, the molecular bonds (-Si-O-Si-) were broken, and the free PDMS molecular chains were covalently grafted onto the surfaces of the particles (Fig. S1)[42]. Repeated siloxane groups (-O-Si-O-) promoted high flexibility in the molecular chain, resulting in a smooth coating surface, and the silanized particle surface became omniphobic and could effectively repel both water and alkanes[43], [44]. In the second step, to form a lubricating coating on the surface of the particles, silicone oil was chosen as the lubricating fluid based on its strong chemical affinity to the PDMS chain grafted onto the surface as well as its excellent chemical stability and low environmental impact[40]. In our experiments, the PDMS-functionalized slippery nanoparticles (SNPs) were redispersed in silicone oil overnight to form a stable lubricating layer on the surfaces of the particles. The PDMS-grafted surface was preferentially wetted by the silicone oil, and the mesoporous structure of the particles may have provided a larger active area and interface to stabilize the lubricating fluid. The particles were finally separated from the lubricant solution by centrifugation and stored under ambient conditions for future applications.

**Fig. 2:**
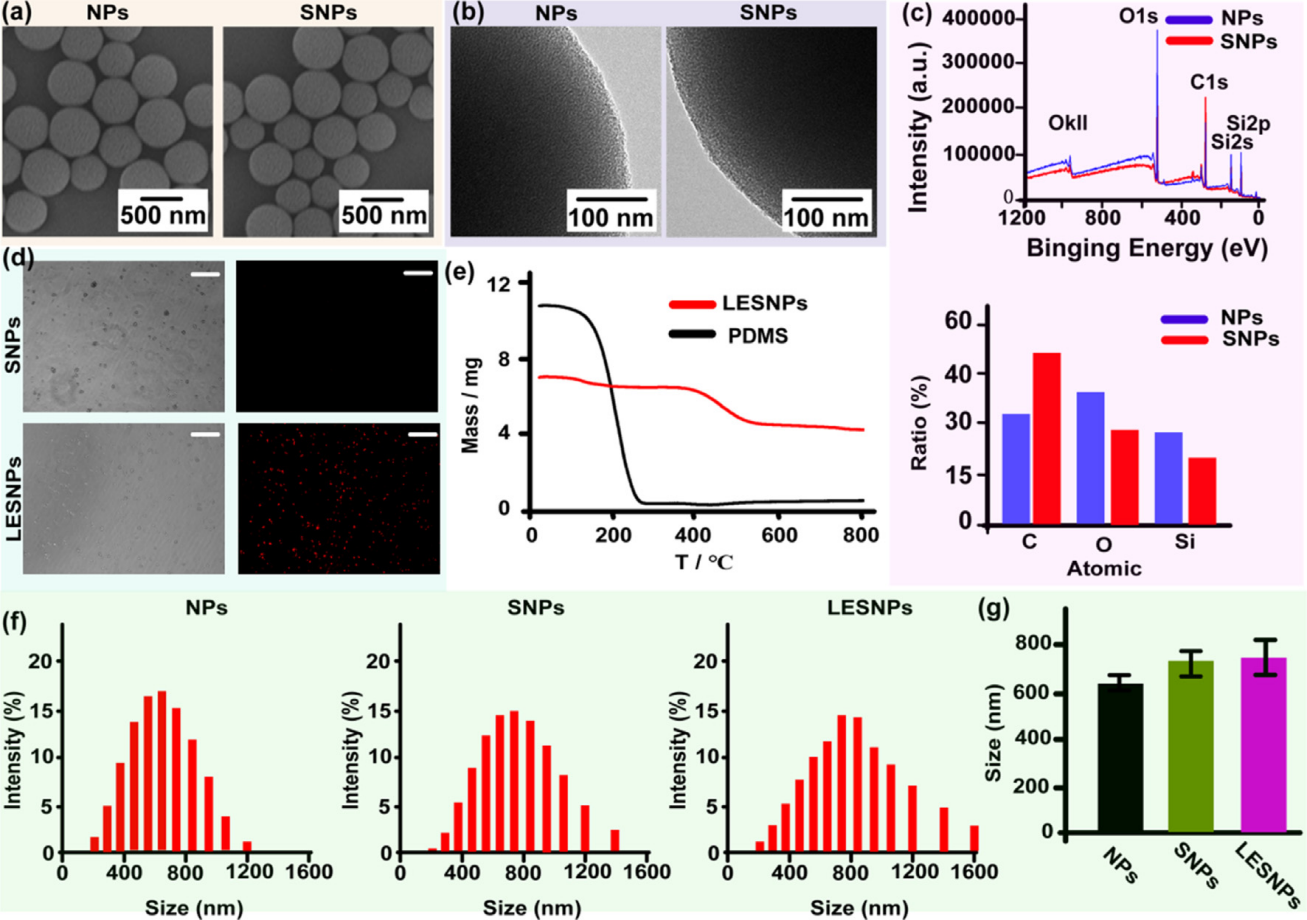
(a) and (b) SEM and TEM images of mesoporous silica nanoparticles (NPs) and “liquid-like” PDMS chains functionalized slippery nanoparticles (SNPs). No significant changes in terms of particles size, shape and surface morphology between SNPs and NPs. (c) XPS spectra of NPs and SNPs surface. After PDMS grafted, the increase of carbon-to-oxygen ratio was observed. (d) Optical and fluorescence microscopy images are showing the SNPs and LESNPs. The liquid layer was stained with fluorescent dye, Rhodamine B. Scale bar: 10 μm. (e) TGA curves of PDMS liquid and LESNPs. (f) Hydrodynamic size distribution of different nanoparticles including NPs, SNPs and LESNPs. (g) Changes in particle size of NPs after grafted with PDMS (SNPs) chains and further PDMS liquid entrenched (LESNPs). Error bar represents the mean ± s.e.m.. N = 3.

The characteristics of the slippery nanoparticle (SNP) microscopic surface were analyzed by scanning electron microscopy (SEM) and transmission electron microscopy (TEM) and are shown in Fig. 2a and 2b. Based on SEM and TEM images, compared with the original mesoporous particles, the size, shape, and surface morphology of the SNPs did not show significant changes. Whether the PDMS chains had been successfully grafted onto the surface of the particles was further investigated and confirmed by X-ray photoelectron spectroscopy (XPS). A glass slide was employed as a silica nanoparticle substitute for modified surface analysis with the same liquid silicone oil coating protocol. The XPS images showed that the carbon-to-oxygen ratio increased (from 0.83 to 1.80) after the PDMS was modified, suggesting that PDMS chains had been successfully grafted onto the silica surface (Fig. 2c). To further verify the lubricating layer entrenchment on the slippery nanoparticles, we attempted to observe the coating by specifically labeling the lubricating layer with a fluorescent dye. The silicone oil was stained with rhodamine B, and then the smooth particles were entrenched and analyzed by fluorescence microscopy. As shown in Fig. 2d, in the fluorescence image, a uniform bright red color was observed on the surfaces of the particles after entrenchment by the lubricating layer, suggesting that the lubricant oil had been successfully immobilized on the SNP surfaces.

We further conducted thermogravimetric analysis (TGA) to examine the amount of lubricant oil immobilized on the particle surface, as shown in Fig. 2e. The TGA images showed weight loss of the LESNPs at the two temperatures of 180 °C and 410 °C, which was probably attributed to the evaporation of the lubricant oil from the particle surfaces and mesopores. The results showed that the weight loss ratios of the LESMPs were approximately 1/6 and 1/4 at the two temperatures, respectively, suggesting that the lubricating oil fixed on the LESMPs accounted for approximately 1/3 of the total weight, and the lubricant on the mesoporous surface accounted for a higher proportion. Additionally, to precisely evaluate the thickness of the lubricating layer on the LESMPs, dynamic light scattering (DLS) was used for characterization. The hydrodynamic size distribution diagram showed that the average diameter of the particles increased slightly after coating with the lubricant (Fig. 2f), indicating that the lubricating oil had evenly coated the particle surface. Further statistical results showed that the average diameter of the particles increased from 735 nm (SNPs) to 830 nm (LESNPs) (Fig. 2g), suggesting that the thickness of the lubricant layer coating the particle surfaces was only tens of nanometers.

### Characterization of anti-adhesion properties of LESNPs

**Fig. 3:**
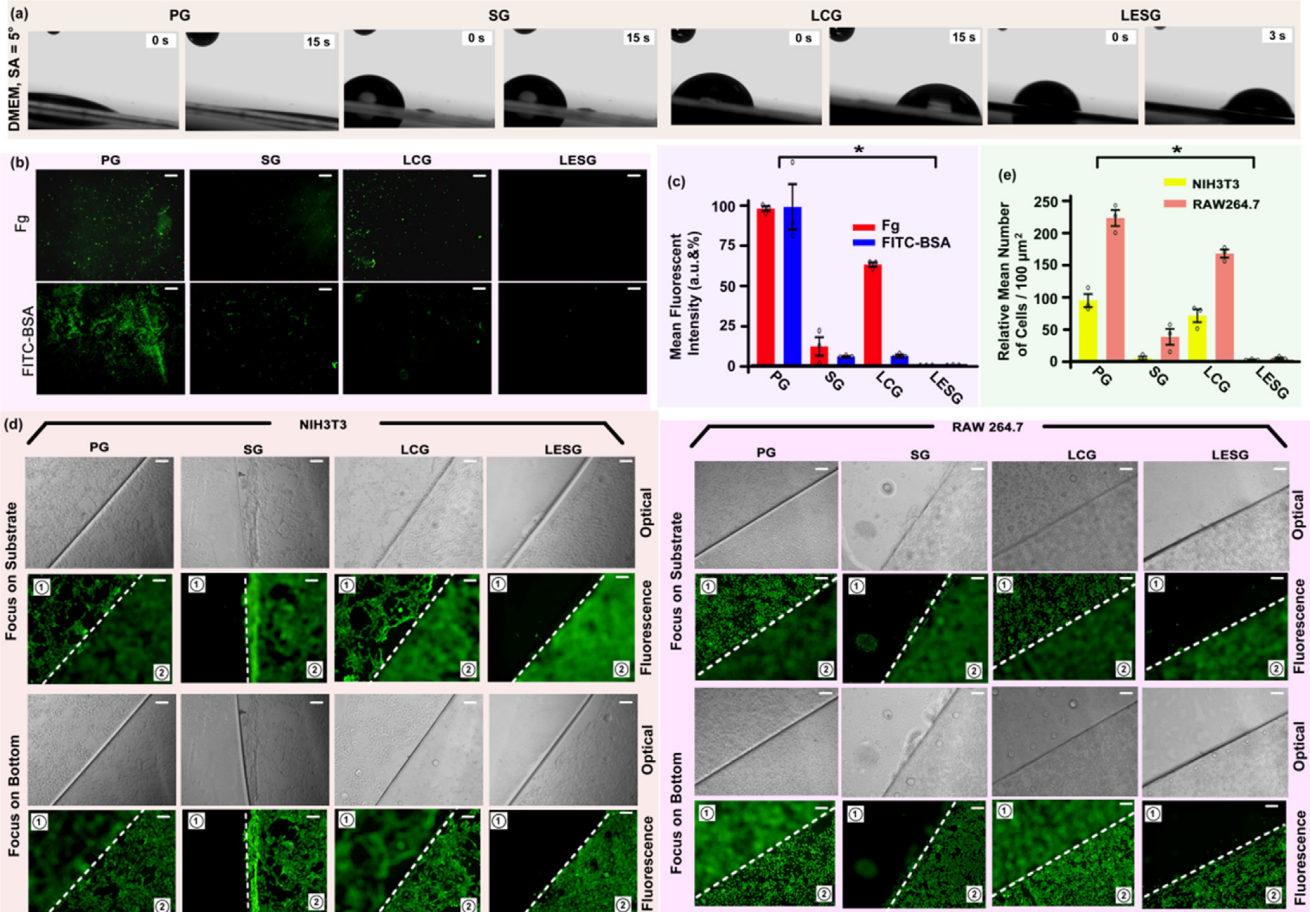
(a) Photographs are showing the slippery behavior of different substrates with sliding angles ∼5°. (b) Fluorescence images and (c) statistical analysis are showing the adhesion of fluorescent FITC-BSA and fibrinogen on different substrate surfaces. (d) Optical and fluorescence images and (e) statistical analysis are showing the adhesion of NIH3T3 cells and RAW264.7 cells (both with green fluorescence) on different substrate surfaces. The sample substrate was indicated as the area “1”, and the bottom surface of the well was indicated as the area “2”, and the substrate boundary was highlighted with dashed line in the merged images. The optical microscopy image and fluorescence image were separately shown. Scale bar: 30 μm. Error bar represents the mean ± s.e.m. * indicates statistically significant compared to the control group (PG) at the level of p < 0.05 using ANOVA followed by a post hoc test. N = 3. (For interpretation of the references to color in this figure legend, the reader is referred to the web version of this article.)

As a important indicator of anti-fouling performance, the slippery behavior of LESG was characterized by using a protein-rich cell culture fluid, DMEM with FBS, as a representative biological fluid. The LESG surface was highly slippery to DMEM and showed no residue at a sliding angle (SA) of ∼ 5° (Fig. 3a). In comparison, the PG was wetted and severely contaminated, while the only surface-grafted SG did not exhibit slipperiness, and on the only surface-coated LCG, fluids took longer to slide down (approximately 5 times longer to cover the same distance). The results indicated that the LESS coating possessed improved slippage and dewettability properties and was hypothetically resistant to proteins and cells. According to the results, the unique dewetting performance of LESSs may benefit from the dual effects of PDMS polymer grafting and coating. The lubricating layer was stabilized on the substrate surface due to the grafting of the PDMS polymer, which can effectively inhibit the surface adhesion.

Protein adsorption onto the surfaces of nanocarriers usually results in uptake by macrophages and other phagocytic systems, which greatly affects the efficiency of drug delivery[14]. FITC-conjugated bovine serum albumin (FITC-BSA, MW ∼ 66 kDa) and fluorescent fibrinogen (Fg) were employed as representative proteins to investigate the resistance of LESG coating-modified substrates to proteins. The two proteins were incubated with LESG for 24 h at room temperature, and the amount of adhesion to LESG was evaluated by fluorescence analysis. To avoid background signals from the fluorescent protein diluent, the solution was replaced with fresh PBS buffer before fluorescence microscopy. In addition, the green fluorescence intensity was quantitatively analyzed by Image J software to indicate the degree of protein adhesion.

The representative fluorescence results of FITC-BSA and Fg on different surfaces are shown in Fig. 3d. After 24 h of incubation, the PG surface exhibited bright green fluorescence in most areas, indicating that the substrate was heavily adhered with proteins including FITC-BSA and Fg. Compared with PG, SG and LCG showed decreased fluorescence intensity, although weak green fluorescence was still observed. In stark contrast, the LESG surface was almost dark and utterly devoid of fluorescence, suggesting the highly efficient inhibition of protein adhesion. The statistical results showed that compared with the PG surface, the surface adhesion of SG was reduced by ∼ 88 % and ∼ 94 % (Fg and FITC-BSA, respectively). The surface adhesion of LCG was reduced by ∼ 35 % and ∼ 92 % (Fg and FITC-BSA, respectively). In comparison, the surface adhesion of the LESG coating was reduced by ∼ 98 % and 97 % (Fg and FITC-BSA, respectively) (Fig. 3e). Our results suggested that due to the formation of a smooth surface and coating with a stable lubricating layer, the dual action significantly inhibited protein adhesion. Achieving an anti-protein adhesion effect on the nanocarrier surface is a critical step in inhibiting uptake by macrophages and the phagocytic system, which is challenging for existing nanocarriers and coating methods. By preventing protein absorption, the LESG coating provides an ideal strategy to eliminate uptake by nanocarriers by macrophages and phagocytic systems.

In addition, the uptake of nanocarriers by phagocytes usually manifests as the adhesion of cells to the nanocarriers, and then pseudopods extend to surround the carrier. Therefore, the inhibition of cell adhesion is also a key indicator for evaluating the anti-uptake effect. Here, fibroblast NIH3T3 cells and macrophage RAW264.7 cells were employed as representative cells to evaluate the cell adhesion resistance of the LESG coating. The non-PDMS-coated substrates, including PG and PDMS chain-grafted SG, and PDMS liquid-coated LCG were utilized as control groups. After 24 h of incubation at 37 °C, cell adhesion was determined by a standard live/dead cell staining assay, and the anti-cell adhesion mechanism was also explored based on cell viability. Live cells were labeled with green fluorescent Calcein AM, and dead cells were labeled with red fluorescent propidium iodide (PI). Since the thicknesses of the substrate and fluorescent cells were not in the same focal plane, fluorescence in the substrate (area “1”) and outside the substrate (area ”2”) was imaged separately, and the boundary between the two areas was highlighted with a dashed line. The fluorescence image showed that both PG and the culture plate were heavily covered by the two types of cells (NIH3T3 and RAW264.7). Compared with PG, the PDMS-grafted surface of SG showed excellent antiadhesion effects toward NIH3T3 cells but could not effectively resist the adhesion of RAW264.7 cells, while LCG did not show significantly improved antiadhesion properties toward either cell type. In stark contrast, the LESG surface exhibited almost no cell attachment (Fig. 3f, S2.1 and S2.2). The adherent cells on different substrates were also statistically analyzed. In terms of the mean numbers of the two types of cells/100 μm^2^, the results of the SG surface were approximately twice lower for NIH3T3 cells and approximately 5 times lower for RAW264.7 cells than those of the PG surface, and for LCG, the results for the two types of cells were less than twice lower than those of the PG surface. The mean numbers of the two types of cells/100 μm^2^ on the LESG surface were less than 3, which were at least 47 times lower for NIH3T3 cells and 73 times lower for RAW264.7 cells than those on the PG surface (Fig. 3g). The results showed that LESG effectively inhibited cell adhesion, benefitting from the dual effects of the smooth interface and the liquid coating. In addition, almost no red fluorescence associated with dead cells was detected in the LESG group, indicating that the LESG coating was nontoxic to cells. These results indicated that the anti-adhesion properties of LESG did not depend on killing cells, but the smoothness and stable mobile nature of the surface of LESG might have prevented cells from attaching to the slippery surface with pseudopodia or other cell adhesion mechanisms, such as agglutinin (a type of protein ligand embedded on the cell membrane). The antiadhesion and biocompatibility of the LESS coating could provide the basis for the application of nanocarriers with antiuptake functionality.

### Characterization of anti-uptake properties of LESNPs *in vitro*

In view of the significance of nanocarrier drugs such as nanoparticles for targeted therapy, the uptake of macrophages hinders the efficient delivery of nanoparticles to diseased tissues, posing a significant barrier to the development of their biological/medical applications[11], [12]. Changing the surface properties of nanoparticles (such as reducing protein adsorption) is expected to alleviate the immunogenicity of nanoparticles and macrophages, inactivate uptake by macrophages and prolong the circulation time[14]. As an effective strategy to prevent nonspecific (protein and cell) adsorption, the liquid-entrenched smooth surface may exert a positive effect on the formation of stealth nanoparticle formulations. Next, the representative macrophage cell line RAW264.7 was employed as a model cell to evaluate the ability of lubricant-entrenched slippery nanoparticles (LESNPs) to evade macrophage uptake. To demonstrate resistance to cell uptake, LESNPs were incubated with RAW264.7 cells at 37 °C for predetermined time intervals (including 2 h, 12 h, and 24 h). At the same time, original nanoparticles without any modification (NPs), “liquid-like” PDMS-grafted slippery nanoparticles (SNPs), and PDMS liquid-coated nanoparticles (LCNPs with PDMS liquid absorbed onto the particle surface without covalent bonding) were utilized as control groups. The uptake of different particles by RAW264.7 cells was assayed by confocal fluorescence microscopy, flow cytometry and optical microscopy. To visualize and quantitatively analyze the cellular uptake of the nanoparticles, various nanoparticles were treated by red fluorescent dye absorption before incubation with the cells. Furthermore, in the confocal experiment, RAW 264.7 cells were labeled with green fluorescence (calcein-AM) for fluorescence imaging, and the number of red particles in the green cells was counted to quantitatively analyze the uptake of different nanoparticles by macrophages.

**Fig. 4:**
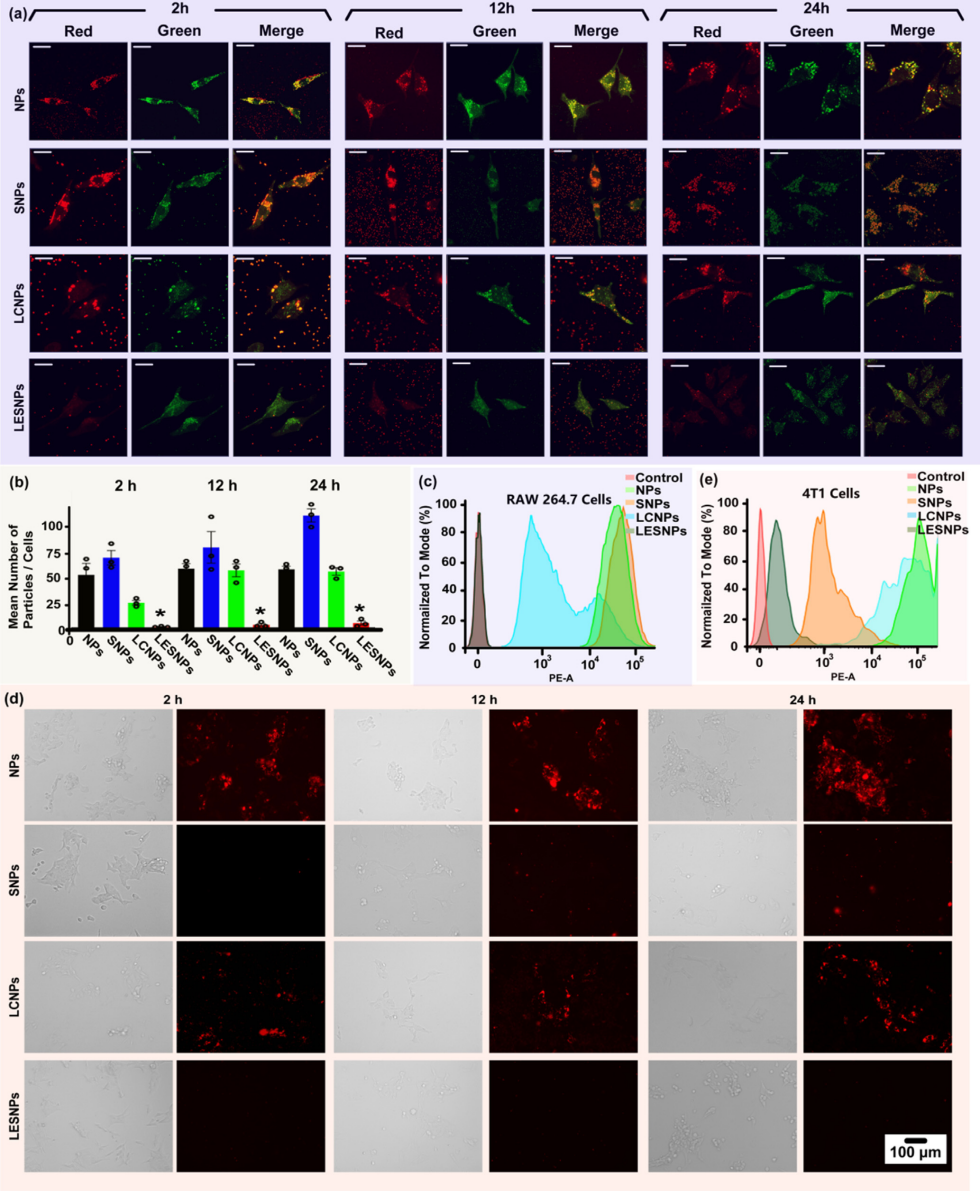
(a) Confocal fluorescence microscopy images are showing the uptake of different nanoparticles including NPs, SNPs, LCNPs and LESNPs by RAW264.7 cells after 2 h, 12 h and 24 h incubation. RAW264.7 cells were labeled with calcein AM (green), nanoparticles were labeled with Rhodamine B (red). Scale bar = 10 μm. (b) Statistical analysis are showing the uptake of different nanoparticles by RAW264.7 cells after 2 h, 12 h and 24 h incubation. After 24 h of incubation, the uptake of LESNPs by RAW cells was significantly inhibited. Error bar represents the mean ± s.e.m. * indicates statistically significant compared to the control groups (NPs, SNPs, and LCNPs) at the level of p < 0.05 using ANOVA followed by a post hoc test. N = 3. (c) Flow cytometry measurement are showing the uptake of different nanoparticles including NPs, SNPs, LCNPs and LESNPs by RAW264.7 cells after 24 h incubation. (d) Fluorescence images are showing the 4T1 cells uptake to different nanoparticles (including NPs, SNPs, LCNPs and LESNPs). Scale bar: 100 μm. (e) Flow cytometry measurement are showing the uptake of different nanoparticles including NPs, SNPs, LCNPs and LESNPs by 4T1 cells after 24 h incubation. (For interpretation of the references to color in this figure legend, the reader is referred to the web version of this article.)

As shown in the confocal fluorescence microscopy images in Fig. 4a, after 2 h of incubation, a large number of red fluorescent NP aggregates appeared in the macrophages, and brighter fluorescence was observed after 24 h, indicating that the mother NPs were extensively taken up by macrophage cells. Compared with the NPs, the number of red fluorescence aggregates for SNPs increased anomalously at all stages (2 h, 12 h and 24 h). The LCNPs showed reduced red aggregates in the short period (<2h) but no significant improvement in the long period (24 h). In contrast to PDMS grafting only or modification by liquid PMDS absorption, liquid-entrenched smooth surface modification induced a distinct reduction in RAW 264.7 cell uptake, and red fluorescence aggregates were almost impossible to identify in the LESNPs even after 24 h of incubation. The statistical results showed that the number of nanoparticles absorbed by a single cell in the LESNP group was no more than four, which was at least 25 times lower than that of the parent nanoparticles (Fig. 4b). Quantitative measurement of the red fluorescence intensity absorbed by the cells using flow cytometry further confirmed this finding (Fig. 4c).

The order of macrophage uptake was not exactly the same as the order of anti-protein and cell adhesion mentioned above, indicating that macrophage uptake may be affected by many factors. According to the results, the uptake of PDMS chain-grafted particles increased, which may have been related to the hydrophobicity induced after modification[45]. In addition, after the free PDMS chains were grafted onto the surfaces of the particles, the flexible silanol bonds may have become more likely to fuse with the phospholipid bilayer on the cell membrane surface to cause increased uptake. The PDMS lubricant layer (LCNPs and LESNPs) played a positive role in inhibiting the uptake[46] of nanoparticles by cells, which might be attributed to the high slipperiness of the flowing liquid coating to the culture medium, which thus prevented cells from attaching to the surface via pseudopods or cell membrane proteins. The LCNPs showed an anti-uptake performance comparable to that of the LESNPs when incubated with macrophages for 0.5 h (Fig. S2), but the LCNP anti-uptake ability was greatly reduced over time. These results indicated that LCNPs only exhibited enhanced anti-uptake performance within a short time, which may have been caused by anti-uptake failure due to the loss of the lubricating fluid. The LESNPs exhibited long-term resistance to cell uptake compared with the LCNPs, indicating that a stable lubricating coating was essential for durable anti-uptake performance.

In addition, to comprehensively evaluate the anti-uptake performance of the modified particles and their drug utilization behaviors, particle uptake by 4T1 cells (mouse-derived tumor cells, representative diseased tissue cells) was further analyzed. All particles, including LESNPs and control groups NPs, SNPs and LCNPs, were labeled with red fluorescent dye and incubated with the 4T1 cells at 37 °C. The uptake of different particles by 4T1 cells was assayed by white-light fluorescence microscopy and flow cytometry. Representative fluorescence images are shown in Fig. 4d. After 24 h of incubation, the LESNPs were almost dark and utterly devoid of red fluorescence compared with the NPs, indicating the highly efficient inhibition of 4T1 cell uptake. Notably, unlike the result for RAW264.7 cells, the SNPs with only “liquid-like” PDMS brushes grafted showed extremely weak red fluorescent aggregates in 4T1 cells, suggesting that PDMS brush-modified particles also showed significantly inhibited uptake by ordinary tissue cells. The LCNPs showed only slight inhibition. The order of the 4T1 cell uptake results was basically the same as that of the protein and cell anti-adhesion results mentioned above, indicating that the inhibitory effects of particles on protein and cell adhesion played a decisive role in the anti-phagocytosis performance toward ordinary tissue cells. The mean percentages of phagocytosed particles in 4T1 cells were quantitatively analyzed by flow cytometry (Fig. 4e). Compared with the NPs, the mean percentage of particles phagocytosed by cells in the LESNP group was less than 20 %. These results further suggested that the nanoparticles might not only benefit from a stable lubricating coating but also resist tissue cell uptake, implying that drug-loaded LESNPs can release drugs into diseased tissues through diffusion rather than endocytosis.

### Evaluation of LESNPs as drug carriers *in vitro* and *in vivo*

After investigating the anti-uptake properties of the nanoparticles, the drug release behaviors and effect performance were studied. First, the water-soluble dye rhodamine B and the oil-soluble dye Nile red were employed as representative drugs with different properties to explore the release behaviors of LESNP-loaded drugs. The smooth nanoparticle SNPs were dispersed in PDMS solutions with different drugs dissolved to load the drugs into the nanoparticles. The excess PDMS solution was removed by centrifugation, the nanoparticles were redispersed in PBS solution, and their drug release behaviors were evaluated by absorbance testing. As shown in Fig. S3, the absorbance image showed that only rhodamine B was released slowly in the PBS solution, suggesting that the method based on liquid coating encapsulation of smooth nanoparticles to resist uptake can facilitate the loading and release water-soluble drugs.

**Fig. 5:**
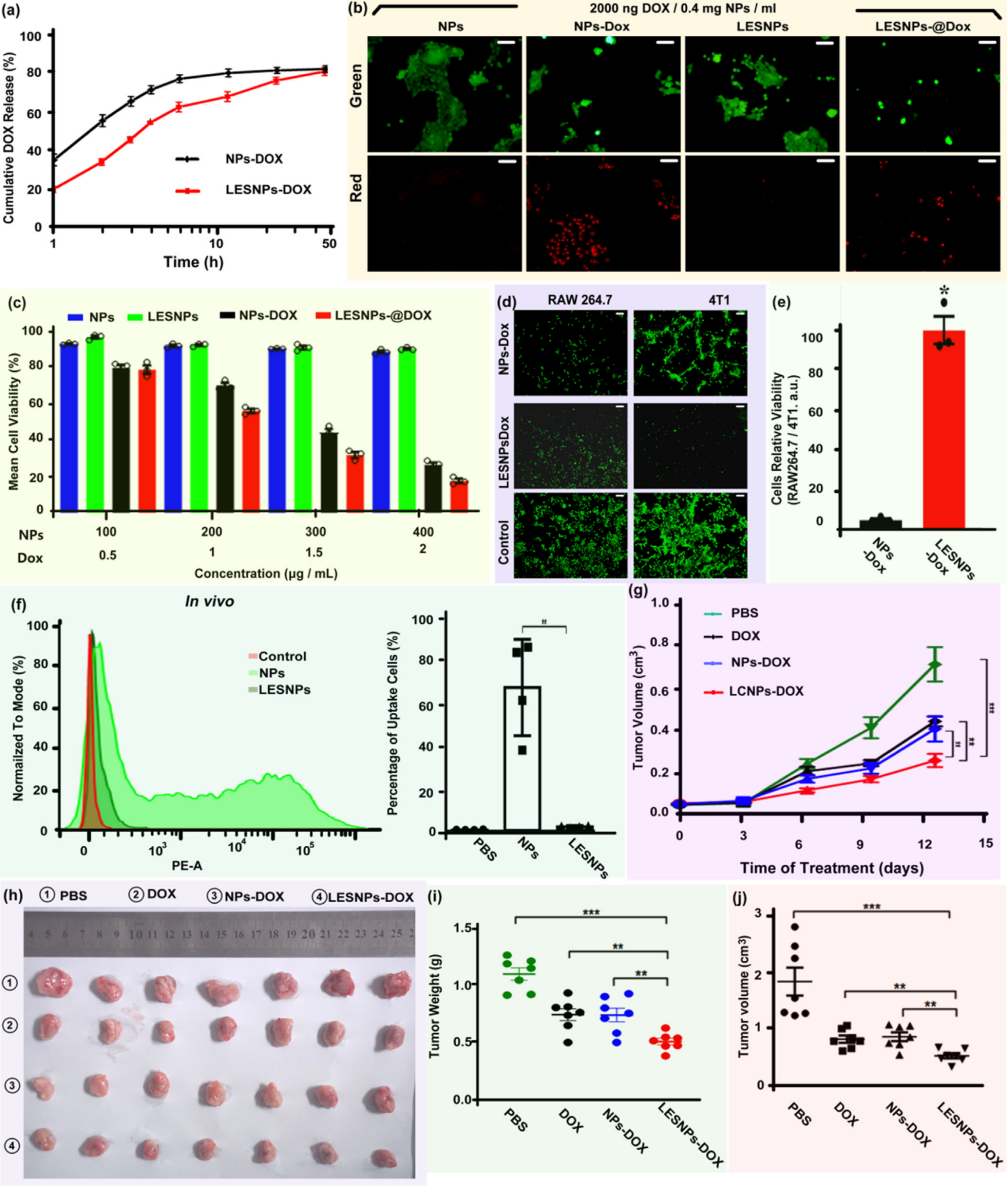
(a) In vitro release of Dox from different Dox-loading nanoparticles including NPs-Dox and LESNPs-Dox in PBS buffers (0.01 M, pH = 7.4). (b) Fluorescence images are showing the viability profile of 4T1 cells (live and dead cells were stained with Calcein AM and propidium iodide, respectively) incubated with different nanoparticles (including no Dox-loading nanoparticles: NPs and LESNPs and Dox-loading nanoparticles: NPs-Dox and LESNPs-Dox) for 24 h. Scale bar = 30 μm. (c) MTT measurement of the viability profile of 4T1 cells incubated with different nanoparticles at different concentrations for 24 h. (d) Fluorescence images are showing the viability profile of RAW264.7 cells incubated with different Dox-loading nanoparticles NPs-Dox and LESNPs-Dox for 2 h and the viability profile of 4T1 cells incubated with remaining different drugs nanoparticles for 24 h. Live cells were stained with Calcein AM. Scale bar = 30 μm. (e) Statistical analysis of cells relative viabilities of RAW264.7 cells and 4T1 cells. (f) The results of the uptake nanoparticles *in vivo* are shown. (g) The 4T1 tumor growth curves from different treatment groups. (h) Excised 4T1 solid tumors from different treatment groups at day 14. (i) Tumor weight from different treatment groups at day 14. (j)Tumor volume from different treatment groups at day 14. Error bar represents the mean ± s.e.m. Statistical significance was determined by analysis of variance (ANOVA) with a post hoc test. NS represents non-significance. *p < 0.05, **p < 0.01, ***p < 0.001.

Subsequently, water-soluble doxorubicin (Dox) was employed as a functional drug to evaluate the drug loading and release behavior of LESNPs and the associated effect performance. Two types of nanoparticles, the original NPs and PDMS-grafted smooth nanoparticles (SNPs), were dispersed in Dox-deionized water and Dox-PDMS solution, respectively. After 24 h of stirring, the drug-loaded nanoparticles (including NPs-Dox and LESNPs-Dox) were separated from the water or PDMS solution by centrifugation and stored at room temperature for future applications. Drug loading was determined by measuring the absorbance of Dox characteristic peaks (480 nm). Representative absorbance images are shown in Fig. S4. Compared with NPs (17 %), the loading percentage of Dox in LESNPs determined by fluorescent absorbance was slightly higher (28 %), which may be attributed to the lubricating layer of LESNPs dissolving the drug. In addition, the *in vitro* drug release profile of Dox-loaded nanoparticles was determined at 37 °C in PBS buffer (0.01 M, pH = 7.4). A representative drug release profile is shown in Fig. 5a. Approximately 79 % of the total loaded Dox was released from LESNPs-Dox within 48 h, which was equivalent to the result for NPs-Dox (∼80). Notably, compared with NPs-Dox, the release rate of LESNPs-Dox was relatively slow, which would have a positive impact on the long-lasting drug effect. Collectively, these results demonstrated that modification with PDMS to form functionalized nanoparticles (LESNPs) is more suitable for drug loading and release.

To further investigate the *in vitro* cytotoxicity of LESNPs-Dox, murine-derived cells (4T1 cells) and human-derived cells (HeLa cells and MCF7 cells) were employed as representative cells and incubated with LESNPs-Dox. Meanwhile, NPs-Dox were employed as a control group. In addition, noncytotoxic nanocarriers are desirable for reducing side effects in biological systems. To eliminate the influence of the nanocarriers on cytotoxicity, the cells were also separately incubated with nonloaded Dox nanoparticles (NPs and LESNPs). After 24 h of incubation at 37 °C, cell viability was analyzed by a standard live/dead cell staining assay and a 3-(4,5-dimethyl thiazol-2-yl)-2,5-diphenyltetrazolium bromide (MTT) assay. Live cells were labeled with green fluorescent Calcein AM, and dead cells were labeled with red fluorescent propidium iodide (PI). As shown in Fig. 5b and Fig. S5a and S5c, for all tested cells (4T1, HeLa and MCF7 cells), sparse red fluorescent cells were observed for NPs and LESNPs, suggesting that both NPs and PDMS-modified LESNPs were nontoxic to all cell lines even at concentrations of up to 400 μg/mL. In contrast, large numbers of red cells were observed in the NPs-Dox and LESNPs-Dox groups, suggesting significant inhibition of cell growth when the cells were treated with Dox-loaded nanoparticles. According to the results of MTT determination, Dox-loaded nanoparticles, including LESNPs-Dox and NPs-Dox, exhibited significantly enhanced cytotoxicity with increasing Dox concentrations. At all studied Dox concentrations, the cytotoxic efficacy of LESNPs-Dox was comparable to that of NPs-Dox (Fig. 5c and Fig. S5b and S5d). In addition, by calculating the half-maximal inhibitory concentration (IC50) value of LESNPs (IC50: 1.26 μg/mL) on 4T1 cells, it was also comparable to the original drug-loaded microparticles NPs-Dox (IC50: 1.44 μg/mL) (Fig. S5e). The results suggested that the nanoparticles could also be used for drug delivery after modification with PDMS (LESNPs).

The key goal in drug delivery to treat tumors is to efficiently deliver drugs into cancer cells to inhibit cell growth and ultimately lead to cell apoptosis[47]. However, it is difficult to achieve the expected effective delivery with most drug carriers, generally because nanocarriers undergo immune phagocytosis (e.g., macrophage uptake) after injection into a living body, preventing their interaction with tumor cells. As an effective strategy to prevent macrophage uptake, LESS coating modification technology may have a positive impact on drug delivery by nanocarriers. Here, RAW264.7 and 4T1 cells were used as representative macrophages and tumor cells, respectively, to examine whether LESNPs could enrich drugs in tumor cells. LESNP-Dox was first incubated with RAW264.7 cells for 2 h, and then the drugs were extracted and incubated with 4T1 cells. Meanwhile, the original NPs were employed as a control group. After 24 h of incubation, the live RAW264.7 and 4T1 cells were labeled with green fluorescent Calcein AM. Cell viability was analyzed by fluorescence microscopy, and the relative cell viability of RAW264.7 and 4T1 cells was used to evaluate the positive effect of the LESS coating on nanoparticle drug delivery. Based on the fluorescence images (Fig. 5d), RAW264.7 cells in the NP group exhibited a small number of green fluorescent cells, and more 4T1 green fluorescent cells were observed, indicating that it is difficult to efficiently aggregate tumor cells by relying on these nanocarriers to deliver drugs. In stark contrast, more green fluorescent RAW264.7 cells than 4T1 cells were observed in the LESNP group, suggesting that the LESS-coated modified nanoparticles inhibited drug absorption by macrophages and were more concentrated in tumor cells. The statistical results showed that the relative cell viability (RAW264.7/4T1) in the LESNP group was at least 18 times higher than that in the original NP group. This result may be attributed to the fact that the modified particles coated with LESS inhibited macrophage uptake and reduced the rate of drug release, which caused a large amount of drug accumulation in tumor cells. On the other hand, following a similar preparation method to that used for LESNPs-Dox, PDMS coating-modified drug-loaded nanoparticles (LCNPs-Dox) were obtained, and their drug release behavior and relative cell viability were further studied. It was found that the drug release rate of LCNPs was also slower than that of NPs and was comparable to that of LESNPs-Dox (Fig. S6a). However, the relative cell viability in the LCNP group was less than twice higher than that in the NP group (Fig. S6b). These results suggested that the slow drug release caused by the coating only plays a secondary role and that the high-efficiency killing of tumor cells in the LESNP group was dominated by inhibiting uptake by macrophages. In general, nanocarriers might benefit from the LESS coating in resisting macrophage uptake, which usually plays a critical role in drug action on diseased tissues *in vivo*.

Subsequently, the nanoparticles were injected into the abdominal cavities of mice to investigate whether the modified particles could achieve an anti-uptake effect *in vivo*. Macrophages were first induced by the intraperitoneal injection of thioglycolate broth. After 3 days, the fluorescence-labeled LESNPs were injected into the intraperitoneal cavity. PBS and fluorescence-labeled NPs served as negative and positive controls, respectively. After 24 h, macrophages were harvested by peritoneal lavage with cold PBS solution and analyzed by flow cytometry. As shown in Fig. 5f, due to the effect of the LESS coating, the modified nanoparticles could also efficiently inhibit the uptake of macrophages *in vivo*. Furthermore, the drug delivery efficacy of LESNPs *in vivo* was measured as the reduction in tumor volume in 4T1 tumor-bearing mice. Different drugs were intratumorally injected into tumor-bearing tissues in BALB/c mice to investigate whether the LESNPs could enhance the antitumor efficacy *in vivo.* The drug was administered at intervals, the growth of the tumors was measured, and the actual tumor tissue was determined after the experiment was completed. All treatments were well tolerated, as no significant body weight changes were observed after receiving the different drug formulations during the treatment (Fig. S7). Compared with PBS solution, the growth of tumors was significantly inhibited after treatment with different Dox formulations (including Dox solution, NPs-Dox and LESNPs-Dox) (Fig. 5g and 5 h). The tumors treated with LESNPs-Dox exhibited remarkably lower weights (Fig. 5i) and smaller volumes (Fig. 5j) than those treated with Dox and NPs-Dox. The results suggested that as drug carriers, nanoparticles modified with the LESS coating could strengthen the antitumor efficacy, likely benefitting from the inhibition of the uptake by the phagocytic system, which thus allowed more of the drug to act on tumor tissues.

## Conclusion

In summary, we developed a unique strategy of entrenching slippery nanoparticles with a lubricating liquid to produce excellent anti-uptake properties. Nanoparticles were grafted with PDMS brushes to form slippery particles, and the lubricating fluid was stably entrapped, which efficiently prevented protein and cell adhesion and exhibited biocompatibility. Coincubation with RAW264.7 cells and intraperitoneal injection experiments confirmed that the nanoparticles exhibited excellent resistance to macrophage uptake *in vitro* and *in vivo* after modification with the LESS coating. In addition, drug loading and release tests showed that modification with the LESS coating could assist nanoparticles in drug loading and effectively enriching drugs in tumor cells, reducing the toxicity to phagocytes. Moreover, the LESNP drug delivery system significantly improves drug utilization. The technology highlights the superior anti-biofouling property of the LESS coating, which has demonstrated huge potential for imparting excellent anti-uptake properties to nanoparticles. We anticipate that the innovative slippery LESS coating will facilitate versatile biological applications and contribute to efficient drug utilization, particularly promoting the clinical transformation of nanomedicine.

## Compliance with Ethics Requirements

*All Institutional and National Guidelines for the care and use of animals were followed.*

## CRediT authorship contribution statement

**Chengduan Yang:** Methodology, Validation, Formal analysis, Investigation, Writing – original draft, Funding acquisition. **Jianming Feng:** Methodology. **Ziqi Liu:** Investigation. **Juan Jiang:** Formal analysis. **Xiafeng Wang:** Formal analysis. **Cheng Yang:** Validation. **Hui-jiuan Chen:** Validation. **Xi Xie:** Funding acquisition, Methodology. **Liru Shang:** Methodology, Formal analysis. **Ji Wang:** Methodology, Funding acquisition. **Zhenwei Peng:** Methodology, Funding acquisition.

## Declaration of Competing Interest

*The authors declare that they have no known competing financial interests or personal relationships that could have appeared to influence the work reported in this paper.*
