# Supplementary material for: Lubricant-entrenched slippery surface-based nanocarriers to avoid macrophage uptake and improve drug utilization
Source: J Adv Res. 2022 Aug 28;48:61–74. doi: 10.1016/j.jare.2022.08.015 (PMC10248789; doi:10.1016/j.jare.2022.08.015)
Supplement: Supplementary data 1 [file mmc1.docx]

**Supporting Information for**

**Lubricant-entrenched Slippery Surface-based Nanocarriers to Avoid Macrophage Uptake and Improve Drug Utilization**

Chengduan Yang^a^, Jianming Feng^b^, Ziqi Liu^b^, Juan Jiang^a^, Xiafeng Wang^a^, Cheng Yang^b^, Hui-jiuan Chen^b^, Xi Xie^a,b^, Liru Shang^a,*^, Ji Wang^a,*^, Zhenwei Peng^a,^*

^a^ The First Affiliated Hospital of Sun Yat-Sen University, Sun Yat-Sen University, Guangzhou, China;

^b^State Key Laboratory of Optoelectronic Materials and Technologies, School of Electronics and Information Technology, Sun Yat-Sen University, Guangzhou, China

*To whom correspondence may be addressed. Corresponding to: Zhenwei Peng, pzhenw@mail.sysu.edu.cn; Ji Wang, [tevacwang@gmail.com](mailto:tevacwang@gmail.com); Liru Shang, shanglr3@mail.sysu.edu.cn

**S1. Supporting information for the illustrating the fabrication procedure of the SNPs.**

**
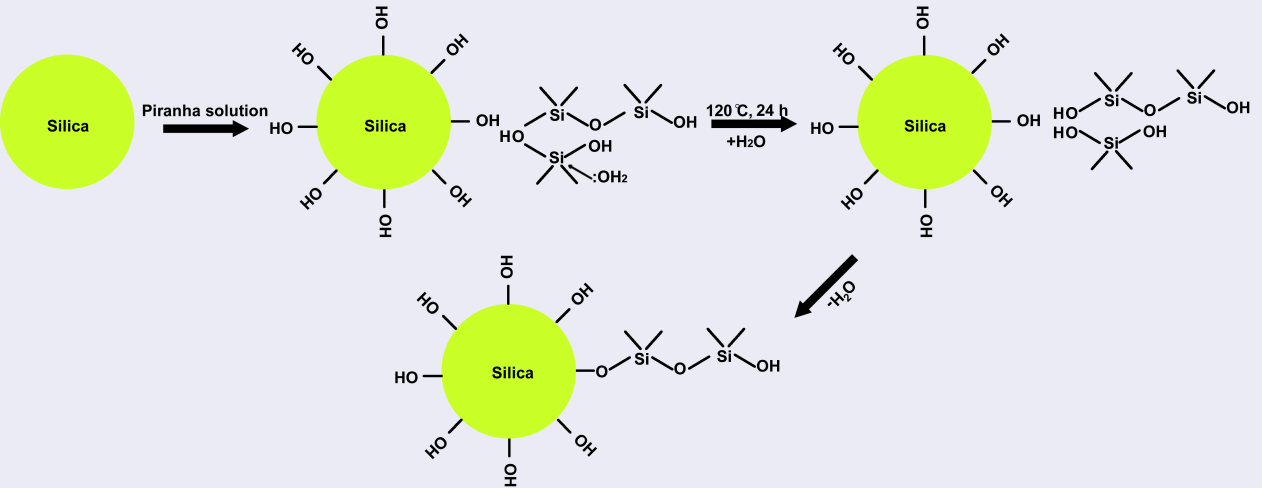
**

**Figure S1** Illustration of the fabrication procedure of the SNPs. PDMS was hydrolyzed and grafted onto the nanoparticles surface under thermally activated condition.^1^ Through the hydrolysis reaction, the -Si-O-Si- bond in PDMS was broken, and only one end was grafted to the nanoparticles surface.

1. Krumpfer, J. W.; McCarthy, T. J. Rediscovering Silicones: "Unreactive" Silicones React with Inorganic Surfaces. Langmuir 2011, 27 (18), 11514-11519.

**S2. Supporting information for the the RAW264.7 cells uptake of different nanoparticles.**

**
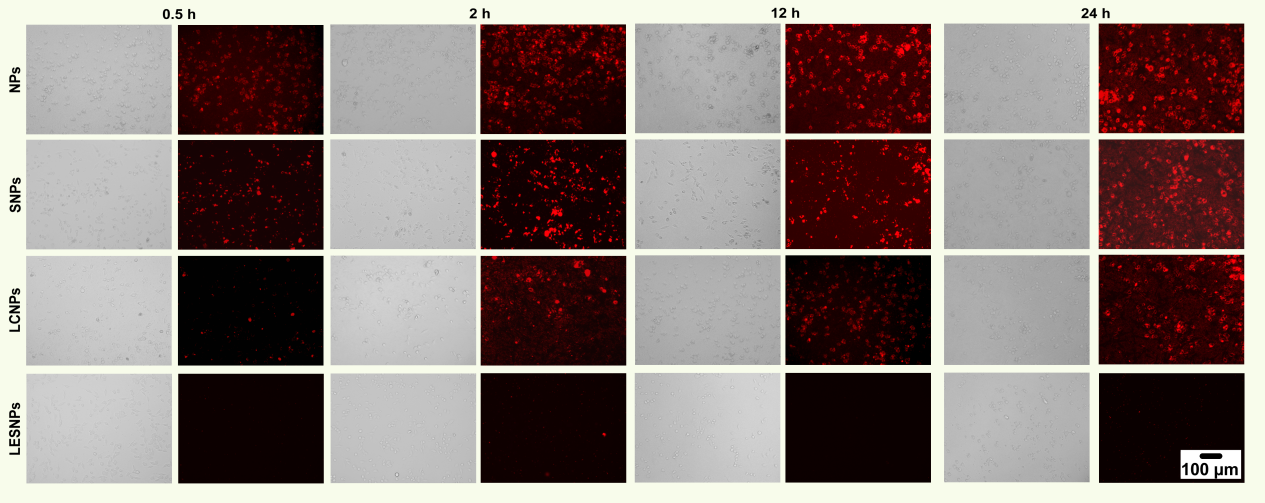
**

**Figure S2.** Optical and fluorescence images are showing the RAW264.7 cells uptake of different nanoparticles (including NPs, SNPs, LCNPs and LESNPs).

**S3. Supporting information for the drug release behavior of LESNPs.**

**
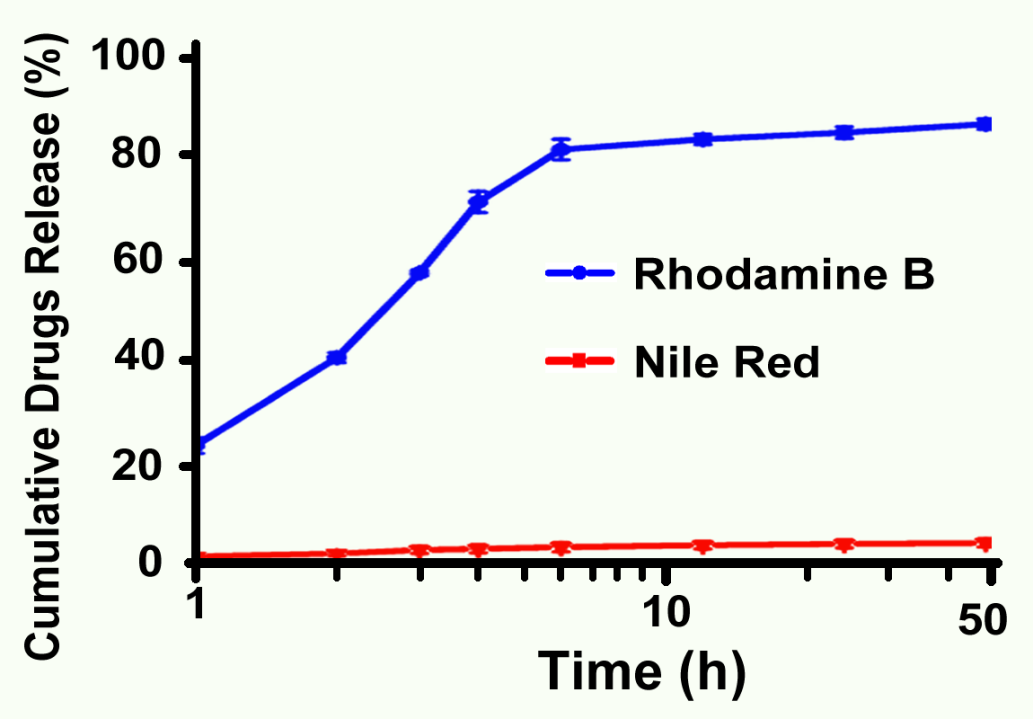
**

**Figure S3.** Cumulative drugs release image are showing the release behavior of LESNPs loaded with different drugs (including water-soluble Rhodamine B and oil-soluble Nile Red) in PBS solution. Water-soluble Rhodamine B can be released continuously, while oil-soluble Nile Red cannot be released. Error bar represents the mean ± s.e.m. N = 3.

**S4. Supporting information for the Dox loading efficiency of different nanoparticles.**


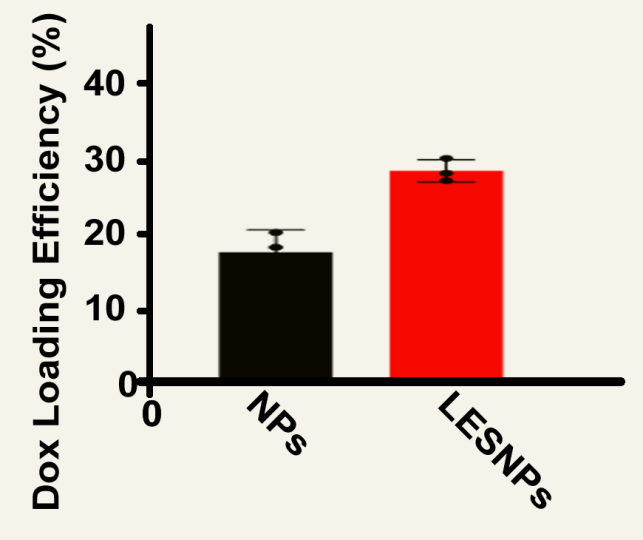


**Figure S4.** Statistical analysis of Dox loading efficiency. Error bar represents the mean ± s.e.m. N=3.

**S5. Supporting information for the cytotoxicity of different nanoparticles in vitro**


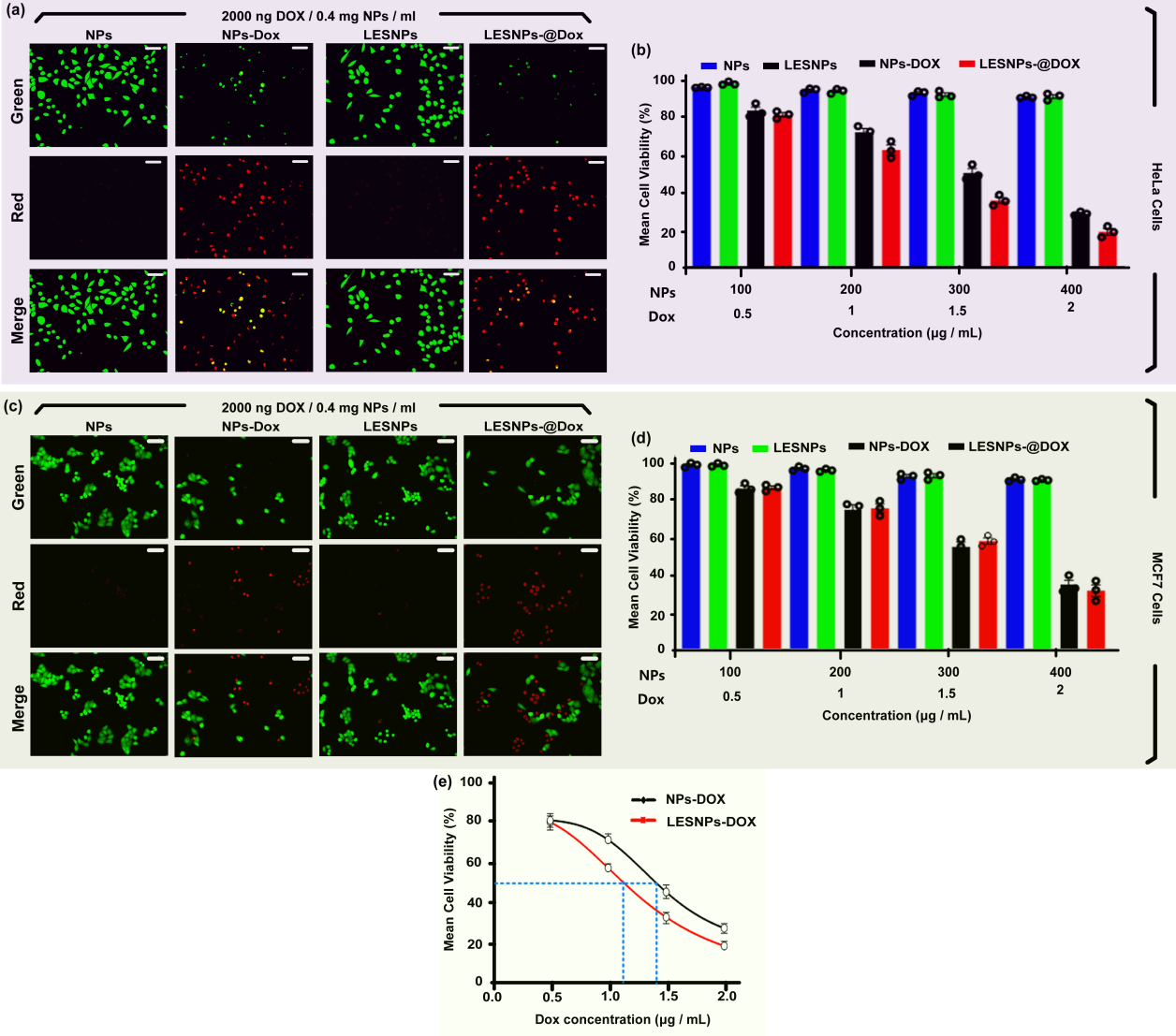


**Figure S5.** (a) Fluorescence images and (b) statistical analysis are showing the in vitro cytotoxicity of different nanoparticles (including NPs, NPs-Dox, LESNPs, and LESNPs-Dox) after incubation for 24 h with HeLa cells. (c) Fluorescence images and (d) statistical analysis are showing the in vitro cytotoxicity of different nanoparticles (including NPs, NPs-Dox, LESNPs, and LESNPs-Dox) after incubation for 24 h with MCF7 cells. (e) Statistical analysis are showing the half maximum inhibitory concentration (IC50) of NPs-Dox and LESNPs-Dox. Scale bar: 30 μm. Error bar represents the mean ± s.e.m. N=3.

**S6. Supporting information for the drug release behavior and cell relative viability of LCNPs-Dox.**


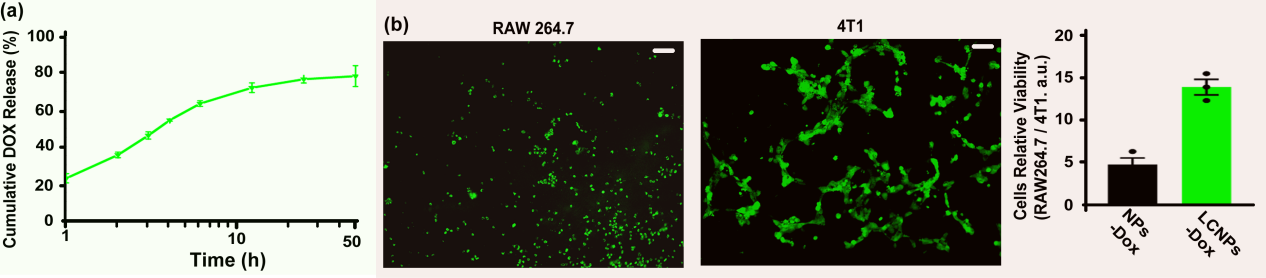


**Figure S6.** (a) In vitro release of Dox from LCNPs-Dox in PBS buffers (0.01 M, pH = 7.4). (b) Fluorescence images and statistical analysis are showing the cells relative viabilities of RAW264.7 cells and 4T1 cells. Error bar represents the mean ± s.e.m. N = 3.

**S7. Supporting information for the *in vivo* applications of different drugs.**


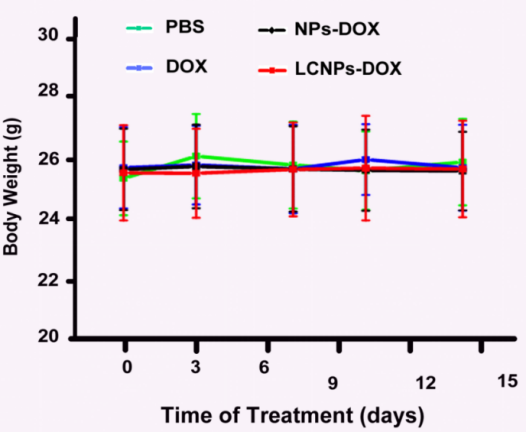


**Figure S7.** The body weight variation of 4T1 tumor-bearing mice during the treatment. Error bar represents the mean ± s.e.m. N=7.

**S8. Supporting information for stability assessment of nanocarriers.**

As the nanocarrier, nanoparticles with persistent anti-uptake properties are highly desirable, yet challenging to achieve with conventional techniques. Here, the stability of the as-prepared LESNPs were explored. The fluorescently labeled LESNPs were statically stored in aqueous solution for 30 days, and the stable existence of the lubricating oil layer was first observed by fluorescence microscopy. Then, the stored LESNPs were incubated with RAW 264.7 for 24 h to explore whether they still exhibited anti-uptake properties. As shown in Figure S8a, the visible red fluorescence still uniformly surrounding particles after 30 days of storage in water, suggested that the immobilization of lubricating oil on the particle surface was stable in aqueous solution. Moreover, after incubation with RAW 264.7, almost no red aggregates were observed (Figure S8b), indicating that LESNPs also had excellent anti-uptake properties after 30 days of storage in water. The above results suggested that LESNPs were stable in aqueous solution.


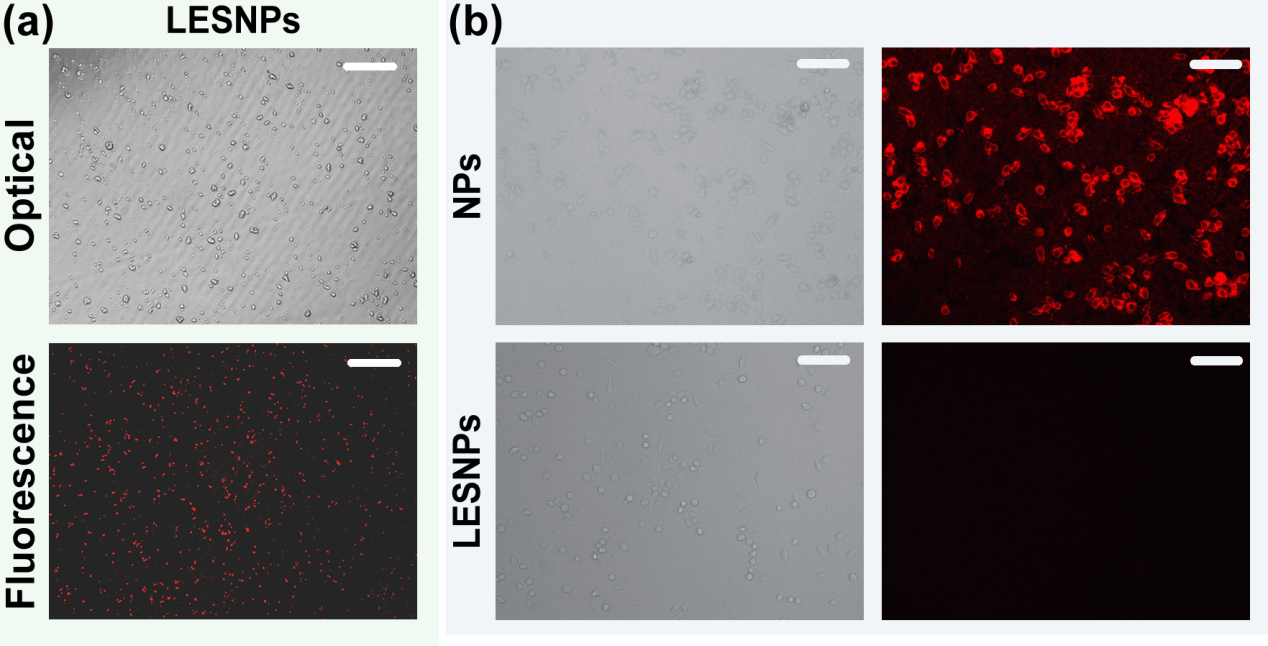


**Figure S8.** (a) Optical and fluorescence images are showing the fluorescently labeled LESNPs still exhibited visible red fluorescence after storage in water for 30 days. Scale bar: 10 μm. (b) Optical and fluorescence images are showing the RAW264.7 cells uptake of different nanoparticles including NPs and LESNPs (storage in water for 30 days). Scale bar: 100 μm.

**S9. Supporting information for absorption spectra of LESNPs.**

*
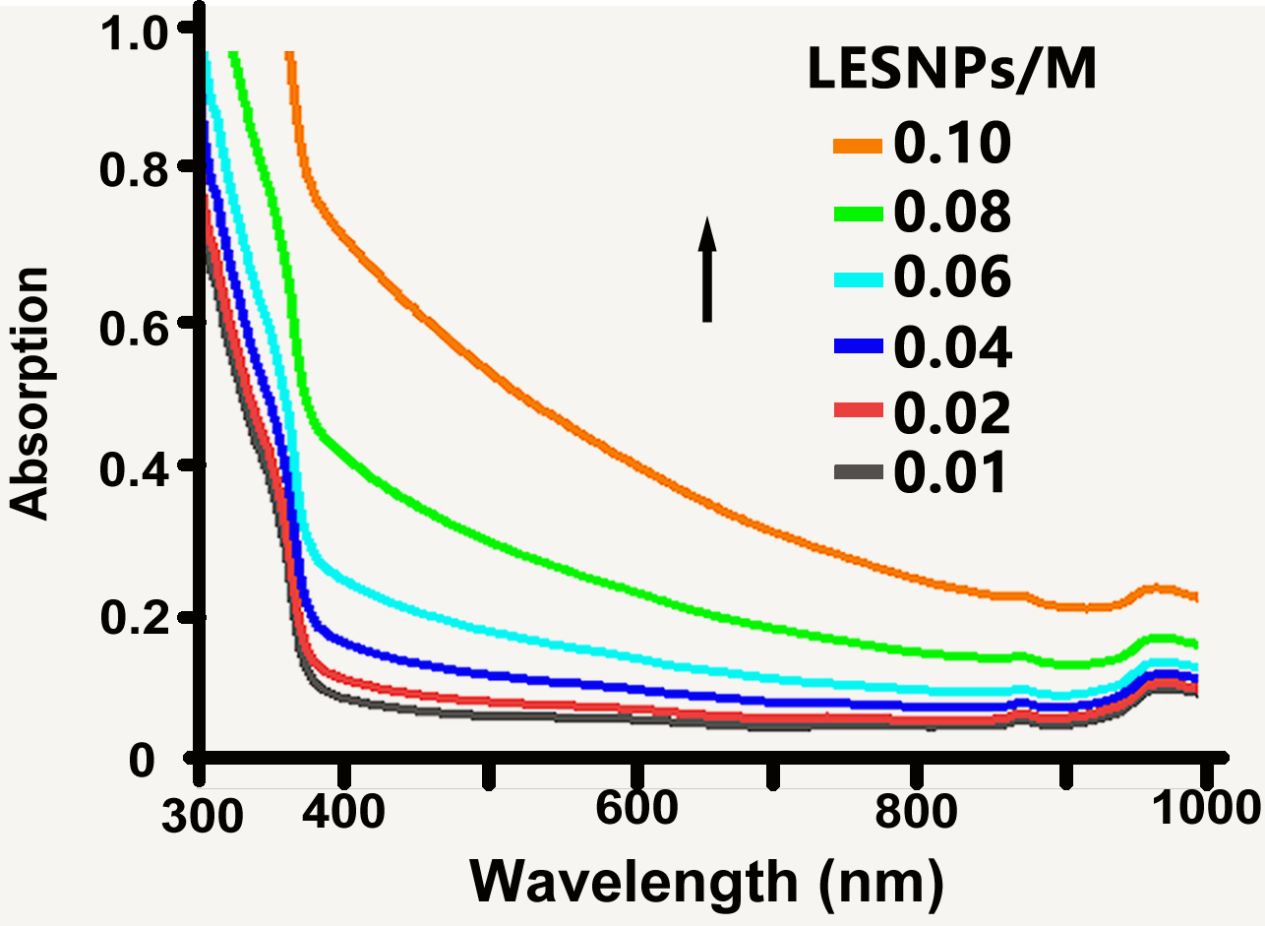
*

**Figure S9.** Absorption spectra of LESNPs in PBS buffer (0.01M, pH = 7.4).

**S10. Supporting information for zeta potential of NPs and LESNPs.**


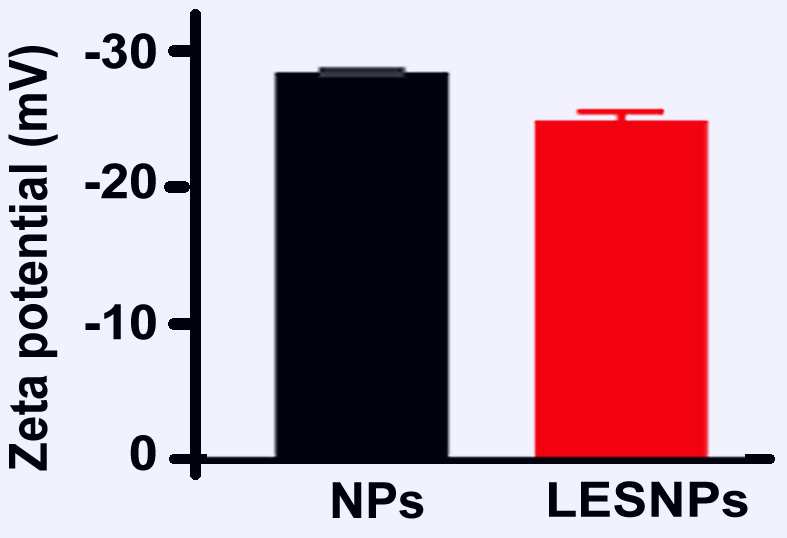


**Figure S10.** Zeta potential of NPs and LESNPs.
